# Introductory editorial to ‘Orgasm: Neurophysiological, Psychological, and Evolutionary Perspectives’

**DOI:** 10.3402/snp.v6.33598

**Published:** 2016-10-25

**Authors:** Victoria Klimaj, Adam Safron

**Affiliations:** Department of Psychology, Northwestern University, Evanston, IL, USA

Few topics in psychology are the source of as much pleasure and anxiety as orgasm. However, the precise functions and evolutionary origins of orgasm remain a mystery. The conditions shaping sexual climax may be particularly complex in humans, whose sexual behavior is characterized by cultural shaping, abstract goals, and frequent non-reproductive motivations.

In light of the fact that ‘nothing in biology makes sense except in the light of evolution’ (Dobzhansky, 1973), we organized this special issue around evolutionary perspectives. At the same time, we recognized that adaptationist hypotheses can be difficult to test, and that biological (perhaps especially psychological) processes are not necessarily evolutionarily advantageous (Gould & Lewontin, 1979). Indeed, given the complexity and flexibility of human sexual behavior, some phenomena may be more fruitfully explored with an agnostic stance toward particular evolutionary models. Accordingly, we were open to a wide range of frameworks and experimental approaches in compiling this special issue, hoping to inspire further research and novel hypotheses.

We have brought together a number of scientists with expertise in the study of orgasm. Our contributors are evolutionary psychologists, animal behavior experts, fMRI researchers, and investigators specializing in the analysis of large-scale surveys. We hoped to address a broad range of questions in this special issue, such as:

1. Why does orgasm intensity and frequency differ between and within individuals?
2. Why is women’s orgasmic capacity more variable (on average) than men’s, with many women reporting difficulty achieving orgasm and some women reporting the ability to experience multiple orgasms?
3. How is the human experience of orgasm and climax different from that of animals?
4. Do orgasms contribute to mate choice, and if so, through what mechanisms do they exert their influences?

Many of the featured papers focus on female orgasm, which reflects broader clinical trends of studying anorgasmia in women, and also ongoing theoretical investigations into whether female orgasm has an evolutionary purpose. Previously, scientists have reached opposing conclusions on the topic of the evolutionary significance of female orgasm, with Lloyd (2006) arguing against female orgasm being a product of natural selection, and others arguing that it has an adaptive function in either mate or sperm selection (Baker, 2006; Dawood, Kirk, Bailey, Andrews, & Martin, 2005; Zietsch, Miller, Bailey, & Martin, 2011). A comprehensive review by Puts, Dawood, and Welling (2012) summarized a variety of studies on the topic, suggesting that the whole of evidence seems to indicate that female orgasm is adaptive rather than vestigial. However, questions remain about the precise effects of orgasm on physiological and psychological processes in human and non-human animals.

## Female orgasmic functioning: evolutionary and relational factors

In this issue, King, Dempsey, and Valentine (2016) provide a high-level review of the debate regarding the function of female orgasm in ‘Measuring Sperm Backflow Following Female Orgasm: A New Method’. They also address the question of mechanism by piloting a novel technique (drawing from farming industry research) for testing women’s sperm retention after orgasm, following suggestions that female orgasm may function to retain sperm and thus increase the possibility of conception by orgasm-inducing males (King & Belsky, 2012). The preliminary data described in this issue indicate that women retain more of a semen-like fluid following orgasm as compared to following a state of sexual arousal without orgasm. King et al. do not propose one particular mechanism by which female orgasm promotes this retention, citing ‘insuck’ as a possibility (King & Belsky, 2012).

In ‘Testing the Mate-Choice Hypothesis of the Female Orgasm: Disentangling Traits and Behaviours’, Sherlock, Sidari, Harris, Barlow, and Zietsch (2016) present the first within-subjects analysis addressing the question of which partner qualities promote orgasm in women, finding that male partners who induce high orgasm rates were rated by their female partners as more humorous, creative, warm, faithful, and better-smelling than partners who had not induced high orgasm rates. Based on these findings, Sherlock et al. argue that female orgasm may be more strongly related to partner traits indicating paternal investment, rather than indicators of physiological fitness. Future work may benefit from employing the within-subject approach used by this study, particularly given its potential for enhanced statistical power and ability to better account for potential confounds between woman’s orgasmic responding and partner characteristics, as well as continue investigating the contribution of different cues to orgasm.

Kontula and Miettinen (2016) further examine questions of orgasmic responding in ‘Determinants of Female Orgasms’, using a large and representative Finnish dataset, covering a wide range of ages (18–81) and timepoints (1979–2015). Orgasm frequency was positively associated with mutual and open approaches to sex as well as positive relationship characteristics. While these results are consistent with existing literature on sexuality and relationships (Klapilovt, Brody, Krejody, Hususdy, & Binter, 2015), Kontula and Miettinen report a particularly surprising finding: orgasm rate did not increase following trends toward greater sexual openness and experimentation across time. That is, though cultural sexual norms over the past several decades contributed to women increasing their numbers of sexual partners and their masturbation frequency, these trends did not correspond to increased orgasmic frequency.

These three studies address questions regarding mate choice and sperm selection, presenting methods and findings that can be expanded in future work. Subsequent investigations may use King et al.’s novel method to examine specific factors that might influence fertility likelihood, such as the desirable personality traits of partners described by Sherlock et al. Whether the same partner characteristics found by Sherlock et al. to be associated with higher rates of orgasm would have been observed in environments of evolutionary adaptation remains unclear. Discrepancies would raise their own intriguing questions, and may potentially be addressed by cross-cultural studies. Finally, a finding highlighted in Kontula and Mietten's paper – of female orgasm rate not increasing despite greater sexual freedom and rates of masturbation – may be of interest to researchers, clinicians, and sex educators, as it may run counter to some intuitions regarding the kinds of practices that might improve sexual sensitivity and responsiveness. However, though Kontula and Mietten's data are of note on a population level, it is yet to be determined how much flexibility and change can be seen throughout individual women’s lifetimes.

## Novel frameworks for understanding orgasm: anatomy, rhythms, and thresholds

In their comprehensive review, ‘The Whole Versus the Sum of the Parts: Toward Resolving the Apparent Controversy of Clitoral Versus Vaginal Orgasms’, Pfaus, Quintana, Cionnaith, and Parada (2016) explore the scientific debate surrounding the location and anatomical origins of female orgasms. Pfaus et al. move beyond a simple dichotomy of vaginal versus clitoral female sexual response, providing a framework thinking about orgasm as an integration of ‘[whole sets] of sensory inputs, movements, body positions, autonomic arousal, and partner- and-contextual-related cues’. Their review suggests that broad differences between vaginal and clitoral orgasm may be only one of many relevant distinctions for understanding differences in experiences of sexuality, which can take myriad forms, all of which can be healthy and rewarding. Finally, they note the importance of considering potential changes to erotic maps and body maps with sexual experience.

In ‘What is Orgasm? A Model of Sexual Trance and Climax via Rhythmic Entrainment’, Safron (2016) proposes a neurophenomenological model for understanding the mechanisms that produce sexual and orgasmic experiences. Safron describes how rhythmic stimulation produces entrainment within nervous systems (and possibly also between partners), resulting in sensory absorption and trance states that culminate in orgasm when critical thresholds are surpassed. The richness of rhythmicity as a source of information about various partner characteristics (such as those indicating investment or genetic fitness) provides a potentially powerful signal of mate quality. While Safron focuses on intrapersonal entrainment, this framework supports a view of sexual interaction that goes beyond a one-sided experience of another’s rhythm. For example, the mutually entraining nature of rhythmic coupling may make it a particularly sensitive gauge for not only partner quality, but also partner complementarity on multiple levels.

In ‘Activation of Sensory and Other Brain Regions in Response to Imagined Versus Physical Genital Stimulation’, Wise, Frangos, and Komisaruk (2016) present fMRI data showing similarities in neural activations for imagined and actual genital stimulation, with both involving sensory cortex and emotion-processing regions of the brain. This both extends previous literature finding overlap between imagined and real sensory experiences (Cichy, Heinzle, & Haynes, 2012) and may suggest a possible neural basis for orgasms that occur in the absence of physical stimulation.

These three papers explore variations in the nature of orgasm-inducing stimulation. Pfaus, Quintana, et al. (2016) provide a novel perspective in focusing on elements of change in orgasmic responding. Further insights may also be obtained by considering the potential malleability of orgasm in males. More speculatively, one may wonder if capacity to experience change in orgasmic experiences might correspond to a capacity to experience fluid attractions, such as those described by Diamond (2008). Safron’s mechanistic model addresses the question of how sexual interaction may (or may not) result in climax based on a model of rhythms allowing for arousal to surpass critical thresholds. Understanding the conditions that allow sexual stimulation to surpass these thresholds may be clinically relevant, with a focus on rhythms potentially enhancing attention and enjoyment during sex. Finally, Wise et al.’s findings may aid in better understanding how sexual perception is impacted by imagination (either via non-reality-based fantasy or envisioning of unfolding sexual experiences as they are happening). These findings may suggest potential benefits from focusing on general attention-capacity and working memory processes in attempting to enhance sexual functioning.

## Animal models and evolutionary approaches: sexual learning and preferences

In ‘Do Rats Have Orgasms?’, Pfaus, Scardochio, Parada, Gerson, Quintana, and Cora-Avila (2016) explore how the presence of orgasm can be established in animals that are incapable of verbalizing their experiences. They review data – including many of their own findings from decades of rigorously detailed experimentation – indicating that rats match the criteria for orgasm-like-responses, including overt physiological processes, short-term behaviors indicating the experience of pleasure, and long-term conditioning as evidenced by place and partner preferences.

In ‘The Role of Orgasm in the Development and Shaping of Partner Preferences’, Coria-Avila, Herrera-Covarrubias, Ismail, and Pfaus (2016) explore the relationship between orgasm and sexual preference-formation in animals. Of particular significance with respect to human orgasm, Coria-Avila et al. describe the pre- and post-ejaculatory factors contributing to male rats forming conditioned sexual preferences. They discuss research by Kippin and Pfaus indicating that a male rat only forms a preference for a female mate if he stays near her in the post-ejaculatory interval. They also review findings demonstrating that disruption to preference-formation occurs when male rat ejaculation occurs with insufficient levels of arousal during copulation. Future work should investigate the degree to which these kinds of dynamics are present in humans and seek to understand how they differ between species and among individuals.

Finally, in ‘An Evolutionary Behaviorist Perspective on Orgasm’, Fleischman (2016) provides a theoretical framework for integrating evolutionary and behaviorist principles. Fleischman emphasizes the importance of considering not only evolutionarily prepared stimuli in contributing to orgasm, but also examining the ways in which orgasms allow organisms to shape each other through conditioning. In addition to describing how organisms can shape each other via providing orgasms (a primary reinforcer), Fleischman goes on to describe the complexity that is possible for reinforcement contingencies. For example, since we are aware of the powerfully reinforcing properties of orgasm, and since many of our goals center around others (e.g. wanting a mate to value us), we are uniquely capable of being reinforced by someone else’s orgasm as a signal that they are sensitive to us (e.g. are capable of being conditioned by us in our pursuit of goals). This sort of high-level shaping demonstrates how behaviorist principles can be just as relevant in humans as they are in other animals.

These final three articles emphasize the change, development, and malleability of sexual responses, and how these factors may be understood through the lens of evolution. Pfaus, Scardochio, et al.’s review of rat data regarding orgasm-like responses is a notable update to our knowledge of animal behavior and may facilitate further evolutionary and developmental work on erotic responding. By further exploring which non-human species have orgasm-like responses – and how these responses are shaped by genetic and experiential factors – we might be able to gain a fuller understanding of the possible adaptive significance of different kinds of orgasmic patterns. Their review also suggests that pleasure is an essential dimension for understanding sexual climax across various species, as the varying levels of reward gained from climax may differ. Coria-Avila et al.’s data incorporate this understanding of orgasm-like-responses in rats to explore sexual preference formation and change; the implications of these studies should be deeply considered in future explorations of human sexuality. Their findings may help to elucidate sexually mediated attachment in humans, which may appear in many forms, ranging from romantic bonds to the development of ‘types’ (and even to fetishistic interests). Finally, Fleischman’s evolutionary behaviorist approach is of particular note when considering human orgasm and demonstrates ways in which evolved mechanisms can exhibit flexibility and context-sensitivity.

## Summary

The papers in this special issue address studies of evolution and physiology, mate choice, sexual functioning and satisfaction, orgasmic plasticity, neurophenomenology, neuroimaging of erotic stimulation and imagination, evidence for and consequences of orgasm-like responses in non-human animals, and sexual conditioning.

As a whole, this collection demonstrates the ways in which orgasm both powerfully shapes behavior and is itself shaped by a wide variety of contexts. These insights extend traditional evolutionary perspectives on sexuality, but do not fall outside the scope of the field if we understand flexibility itself to be an evolved aspect of our natures. Indeed, the wide variety of sexual preferences and manifestations of eroticism in our world can be understood as examples of the very sexual flexibility that allows complex organisms to flourish and reproduce.

We are grateful to Harold Mouras, the editorial team, and the contributors to this special issue of *Socioaffective Neuroscience and Psychology* for their significant work on this collection. These papers present diverse perspectives on a richly complex and multi-faceted phenomenon. We hope this issue will stimulate future research on a topic that is of such great importance to so many people.

*Victoria Klimaj, BA, Guest Editor* Department of Psychology Northwestern University, Evanston, IL, USA
*Adam Safron, MSc, Associate Editor* Department of Psychology Northwestern University, Evanston, IL, USA
